# Effect of Fermentation, Drying and Roasting on Biogenic Amines and Other Biocompounds in Colombian Criollo Cocoa Beans and Shells

**DOI:** 10.3390/foods9040520

**Published:** 2020-04-21

**Authors:** Johannes Delgado-Ospina, Carla Daniela Di Mattia, Antonello Paparella, Dino Mastrocola, Maria Martuscelli, Clemencia Chaves-Lopez

**Affiliations:** 1 Faculty of Bioscience and Technology for Food, Agriculture and Environment, University of Teramo, Via R. Balzarini 1, 64100 Teramo, Italy; cdimattia@unite.it (C.D.D.M.); apaparella@unite.it (A.P.); dmastrocola@unite.it (D.M.); cchaveslopez@unite.it (C.C.-L.); 2 Grupo de Investigación Biotecnología, Facultad de Ingeniería, Universidad de San Buenaventura Cali, Carrera 122 # 6-65, Cali 76001, Colombia

**Keywords:** biogenic amines, polyphenols, histamine, microbiota, roasting

## Abstract

The composition of microbiota and the content and pattern of bioactive compounds (biogenic amines, polyphenols, anthocyanins and flavanols), as well as pH, color, antioxidant and reducing properties were investigated in fermented Criollo cocoa beans and shells. The analyses were conducted after fermentation and drying (T1) and after two thermal roasting processes (T2, 120 °C for 22 min; T3, 135 °C for 15 min). The fermentation and drying practices affected the microbiota of beans and shells, explaining the great variability of biogenic amines (BAs) content. Enterobacteriaceae were counted in a few samples with average values of 10^3^ colony forming units per gram (CFU g^−1^), mainly in the shell, while *Lactobacillus* spp. was observed in almost all the samples, with the highest count in the shell with average values of 10^4^ CFU g^−1^. After T1, the total BAs content was found to be in a range of 4.9÷127.1 mg kg^−1^_DFW_; what was remarkable was the presence of cadaverine and histamine, which have not been reported previously in fermented cocoa beans. The total BAs content increased 60% after thermal treatment *T2*, and of 21% after processing at *T3*, with a strong correlation (*p* < 0.05) for histamine (ß = 0.75) and weakly correlated for spermidine (ß = 0.58), spermine (ß = 0.50), cadaverine (ß = 0.47) and serotonine (ß = 0.40). The roasting treatment of *T3* caused serotonin degradation (average decrease of 93%) with respect to unroasted samples. However, BAs were detected in a non-alarming concentration (e.g., histamine: n.d ÷ 59.8 mg kg^−1^_DFW_; tyramine: n.d. ÷ 26.5 mg kg^−1^_DFW_). Change in BAs level was evaluated by principal component analysis. PC1 and PC2 explained 84.9% and 4.5% of data variance, respectively. Antioxidant and reducing properties, polyphenol content and BAs negatively influenced PC1 with both polyphenols and BA increasing during roasting, whereas PC1 was positively influenced by anthocyanins, catechin and epicatechin.

## 1. Introduction

In the last years, an increase in global cocoa production has been observed with a market demand of high-quality cocoa products [1]. Colombian cocoa was declared by the International Cocoa Organization as “fine” and “flavour” due to the agro-ecological characteristics of the areas in which it is cultivated and the adequate fermentation and drying processes that are carried out. In particular, Criollo is a variety of Colombia and other Latin American countries, known for its high quality.

Cocoa is produced from cocoa beans that undergo several processes such as fermentation, drying, roasting, dutching, conching, and tempering. In the first stages, cocoa pods (fruits) are picked from the trees (*Theobroma cacao*), collected in piles and immediately opened or left to stand for a few days (pod storage) to obtain positive effects on the quality of the final products. After harvesting, beans together with mucilage are removed from the pod, fermented, dried, and roasted [2].

Fermentation is essential for the degradation of mucilage thanks to the production of ethanol, which kills cocoa bean cotyledons, and to the production of different organic acids and important volatile compounds that diffuse into the interior of the beans and react with substances responsible for the flavour of final products during the subsequent roasting process. In addition, fermentation influences some functional properties such as antiradical activity and reduces the power of cocoa beans [3,4]. However, biogenic amines (BAs) can be formed during this step, with detrimental effects on cocoa quality and human health. The occurrence of BAs in food originates from decarboxylation of free amino acids, amination and transamination of ketones and aldehydes or during thermal processes. In fermented products, the concentration of BAs is the result of a balance between formation and degradation reactions in which several microorganisms are involved. In fact, cocoa microbiota may present strains with decarboxylase activity [5,6,7,8] and amino-oxidase activity [9].

A decrease of bioactive compounds, such as BAs and polyphenolic compounds, occurs in different steps of the cocoa beans processing, affecting their final content and functional properties in cocoa derivatives [10,11,12]. During roasting, physical and chemical changes occur in the beans, such as differences in colour, removal of undesirable volatile compounds, formation of desirable aroma and flavour, reduction of water content (up to 2%), and formation of a brittle structure, as well as changes in flavanols, proanthocyanidins and antioxidant activity [13,14]. In addition, peculiar cocoa volatile compounds are generated by Maillard reactions and their release is favoured by modifications of the matrix structure [15]. In spite of this, during roasting critical changes may also take place such as the formation of water-insoluble melanoidins, the degradation of catechin-containing compounds [16], the reduction of polyphenol content and antioxidant activity [17], and an increase of the biogenic amines content [12]. If some Maillard Reaction products, such as melanoidins, are required for the development of the peculiar cocoa sensory characteristics and brown colour, some furanic compounds are supposed to have negative effects on human health, as they can show cytotoxicity at high concentration and are “possibly carcinogenic to humans” [18]. Furthermore, since the presence of cocoa shell in cocoa beans derivatives adversely affects the final product quality [19], beans should be peeled before or after roasting [20,21].

The present research was aimed to study the effect of fermentation, drying and roasting on the microbiological, physical and chemical characteristics of Colombian Criollo cocoa (bean and shell), with a particular focus on the content of bioactive compounds such as BAs and polyphenols.

## 2. Materials and Methods

### 2.1. Origin of the Samples

Criollo cocoa bean samples were collected in spring 2018 directly from 18 farms (identified with a numerical code) located in three Departments of Colombia, with different environmental conditions and different fermentation and drying systems (Table 1). Thirteen samples were from Valle del Cauca, located in the western part of the country, between 3°05′ and 5°01′ N latitude, 75°42′ and 77°33′ W longitude; four samples from Cauca, located in the southwest of the country on the Andean and Pacific regions, between 0°58′54″ N and 3°19′04″ N latitude, 75°47′36″ W and 77°57′05″ W longitude; one sample from Nariño, located in the west of the country (1°16′0.01″ N latitude and 77°22′0.12″ W longitude) and, despite low altitude, affected by cold winds from the south of the continent.

### 2.2. Samples Preparation and Defatting

The samples were collected at the final stage of fermentation and drying (step T1). Moreover, dried cocoa beans were divided into two batches and treated in a convection oven (Memmert UN110, Büchenbach, Germany) in two different commercial roasting conditions: T2, 120 °C for 22 min; T3, 135 °C for 15 min.

After cooling, the shell was removed manually from the cocoa beans. After the removal of the external skin and grinding (IKA M20, Staufen, Germany), cocoa samples were defatted by three cycles of hexane washing (8 g of cocoa sample in 50 mL of hexane), following the method described by Di Mattia et al. [4]. Four grams of sample were weighed and 25 mL of hexane added, then the mixture was vortexed for 1 min and centrifuged (2325× *g* for 10 min), each time discharging the supernatant. To completely remove the hexane from the sample, the lipid-free solids were air-dried at room temperature. The fat-free samples were then used for the extraction of the phenolic fraction and other chemical determinations.

### 2.3. Moisture and pH Determination

The pH of defatted cocoa nibs was measured by diluting in distilled water (1:1) by using an electrode probe connected to a pHmeter (FE20, Mettler Toledo, Columbus, OH, USA).

Moisture content was determined according to the official procedure adopted by the Association of Official Analytical Chemists (AOAC) [22]. In particular, 1 g of sample was dried in a forced-air drying oven at 105 °C up to a constant weight.

### 2.4. Microbiological Analyses

Microbiological analyses were performed according to Chaves et al. [23]. From samples of dried cocoa beans, the beans (from here they are beans without shell) and the shells were obtained by manual separation. Twenty grams of cocoa nibs and separate shells were homogenized in a Stomacher Lab-blender (Thomas Scientific, Swedesboro, NJ, USA) in 90 mL phosphate buffer solution (PBS, Biolife, Milan, Italy) sterile solution, pH 7.4. Decimal dilutions of the suspension were prepared in PBS, plated and incubated as follows: Enterobacteriaceae were counted and isolated in Violet Red Bile Glucose Agar (Oxoid, Basingstoke, UK) at 37 °C in anaerobiosis for 48 h; mesophilic aerobic bacteria in Plate Count Agar (PCA) at 30 °C for 48 h; thermophilic aerobic bacteria in PCA and incubated at 45 °C for 48 h; lactobacilli in De Man Rogose and Sharp (MRS) Broth (Oxoid, Basingstoke, UK) at 37 °C in anaerobiosis for 72 h; lactic streptococci in M17 agar (Oxoid, Basingstoke, UK) at 37 °C in anaerobiosis for 72 h; yeasts in Yeast Extract-Peptone-Dextrose (YPD) agar medium and Walerstein Laboratory (WL) medium agar (Biolife, Milan, Italy) at 25 °C for 48 h; moulds in DG18 Agar (Oxoid, Basingstoke, UK) and Czapec-Agar (Biolife, Milan, Italy) added with 150 ppm chloramphenicol (Sigma-Aldrich Italy, Milan, IT) for 5 days.

### 2.5. Biogenic Amines Determination

Defatted samples were subjected to BAs extraction, detection, identification and quantification by high-performance liquid chromatography (HPLC) using an Agilent 1200 Series (Agilent Technologies, Milano, Italy), optimizing the method described by Chaves-Lopez et al. [23]. Shortly after, 1.0 g of sample was added of 5.0 mL of 0.1 N HCl and stirred in vortex (1 min) and ultrasound (20 min). It was centrifuged (Hettich Zentrifugen, Tuttlingen, Germany) at relative centrifugal force of 2325× *g* for 10 min and the supernatant recovered. Then, 150 µL of saturated NaHCO_3_ was added to 0.5 mL of the supernatant, adjusting the pH to 11.5 with 0.1 N NaOH. For derivatization, 2.0 mL of dansyl chloride/acetone (10 mg mL^−1^) was added and incubated at 40 °C for 1 h under agitation (195 stokes) (Dubnoff Bath-BSD/D, International PBI, Milano, Italy). To remove excess of dansyl chloride, 200 µL of 30% ammonia was added, allowed to stand for 30 min at room temperature, and diluted with 1950 µL of acetonitrile.

In a Spherisorb S30ODS Waters C18-2 column (3 µm, 150 mm × 4.6 mm ID), 10 µl of sample were injected with gradient elution, acetonitrile (solvent A) and water (solvent B) as follows: 0–1 min 35% B isocratic; 1–5 min, 35%–20% B linear; 5–6 min, 20%–10% linear B; 6–15 min, 10% B isocratic; 15–18 min, 35% linear B; 18–20 min, 35% B isocratic. Identification and quantification of cadaverine (CAD), dopamine (DOP), ethylamine (ETH), histamine (HIS), 2-phenylethylamine (PHE), putrescine (PUT), serotonin (SER), spermidine (SPD), spermine (SPM), and tyramine (TYR) was performed by comparing retention times and calibration curves of pure standards. The results were reported as mg of BA kg^−1^ of defatted dry weight (of DFW).

### 2.6. Colour Analysis

Colour analysis of the cocoa samples was carried out by a Minolta Bench-top Colorimeter CR-5 (Konica Minolta, Tokyo, Japan) CM-500 spectrophotometer. Before analysis, two calibrations were carried out, one with black standard and the other one with white standard. For each measurement, a single layer of grounded cocoa beans was spread on a Petri dish. The analysis was repeated three times on each sample. 

The instrument gave the results in terms of CIE L* a* b* parameters (CIELAB is a colour space specified by the International Commission on Illumination, French Commission Internationale de l’éclairage, CIE), where L* indicates the lightness within the range from 0 (black) to 100 (white); a* ranges from −60 (green) to +60 (red); b* ranges from −60 (blue) to +60 (yellow). a* and b* indicate colour direction and from these values we obtained the Hue angle (h°), calculated as h° = arctan(b*/a*) 

### 2.7. Anthocyanin Determination

The total anthocyanins content was determined by the method described by do Carmo Brito et al. [11]. In brief, 1.0 mL of 95% ethanol and 1.5 N hydrochloric acid solution (85:15 *v*/*v*) were added to 0.1 g of defatted cocoa sample, then stirred in vortex for two min and allowed to stand overnight. The sample was centrifuged at 10,000× *g*, and the supernatant was suitably diluted to measure its absorbance at 535 nm by a spectrometer (Eppendorf Biospectrometer kinetic, Hamburg, Germany. The results were reported as mg of anthocyanins g^−1^ of sample.

### 2.8. Extraction of the Phenolic Fraction

The defatted samples were further ground with mortar and pestle to reduce the powder size and to allow better contact of the extracting solvent with the sample. The sample extraction was carried out according to Di Mattia et al. [4] with some modifications. One gram of defatted sample was added to 5 mL of 70:29.5:0.5 acetone/water/acetic acid; the mixture was vortexed for 1 min, then sonicated in an ultrasonic bath (Labsonic LBS 1, Falc, Treviglio, Bergamo, Italy) at 20 °C for 10 min and finally centrifuged (2325× *g* for 10 min). The surnatant liquid was recovered and filtrated through cellulose filters. The extracted polyphenols were then stored in the freezer at −32 °C until analyses. This extract was used for the evaluation of total polyphenol content, radical scavenging activity and ferric reducing properties. For flavanols analysis, samples were extracted, kept at −80 °C and analysed on the same day or at the latest a few days after extraction.

#### 2.8.1. Total Polyphenols Content (TPC)

The total polyphenol content (TPC) was determined according to a procedure modified from Di Mattia et al. [10]. To a volume of 0.1 mL of diluted defatted sample extract, water was added up to a volume of 5 mL, and 500 μL of Folin–Ciocalteu reagent was added. After 3 min, 1.5 mL of a 25% (*w*/*v*) Na_2_CO_3_ solution was added and then deionized water up to 10 mL of the final volume. Solutions were maintained at room temperature under dark conditions for 60 min and the total polyphenols content was determined at 765 nm using a spectrophotometer (Lambda Bio 20 Perkin Elmer, Waltham, MA, USA) Gallic acid standard (Fluka, Buchs, Switzerland) solutions were used for calibration. Results were expressed as milligrams of gallic acid equivalents (GAE) per gram of defatted and dry weight.

#### 2.8.2. Flavanols Identification and Quantification

HPLC (high-performance liquid chromatography) was used for separation, quantitative determination and identification of flavonoids. The chromatographic analyses were performed on a 1200 Agilent Series HPLC (Agilent Technologies, Milano, Italy) equipped with a quaternary pump, a degasser, a column thermostat, an autosampler injection system and a diode array detector (DAD). The system was controlled by Agilent ChemStation for Windows (Agilent Technologies). Flavanols determination was carried out according to Ioannone et al. [13]. The sample (20 µL) was injected into a C18 reversed-phase column. Separation of phenolic compounds was carried out at a flow rate of 1 mL/min with a non-linear gradient from A (1% acetic acid solution) to B (ACN). Gradient elution was as follows: from 6% to 18% B from 0 to 40 min, from 18% to 100% B from 40 to 45 min, from 100% to 6% B from 45 to 50 min, isocratic from 50 to 53 min. The DAD acquisition range was set from 200 to 400 nm. The calibration curves were made with epicatechin and catechin, and the results were expressed as mg per gram of defatted and dry weight.

#### 2.8.3. ABTS (2,2′-azino-bis(3-ethylbenzthiazoline-6-sulfuric acid)) Assay

The radical scavenging activity was measured by ABTS (2,2′-azino-bis (3-ethylbenzthiazoline-6-sulfuric acid)) radical cation decoloration assay, as described by Re et al. [24]. The ABTS radical stock solution was prepared by dissolving ABTS in water to a 7 mM concentration and by making this solution react with 2.45 mM of potassium persulfate. The mixture was then left in the dark at room temperature for 12–16 h before use. The ABTS+• stock solution was diluted in water to an Abs of 0.70 ± 0.02 for the analysis, and the reaction was started by the addition of 30 µL of cocoa extract to 2.97 mL of ABTS+• radical solution. The bleaching rate of ABTS+• in the presence of the sample was monitored at 25 °C at 734 nm using a spectrophotometer (Lambda Bio 20, Perkin Elmer, Boston, MA, USA) and decoloration after 5 min was used as the measure of antioxidant activity. Radical scavenging activity was expressed as Trolox Equivalents Antioxidant Capacity (TEAC-µmol of Trolox equivalents per g of defatted and dry weight), calculated by the ratio between the correlation coefficient of the dose–response curve of the sample and the correlation coefficient of the dose–response curve of Trolox, the standard compound.

#### 2.8.4. Ferric Reducing Antioxidant Power (FRAP)

The reducing activity of the samples was determined according to the method described by Benzie and Strain [25] with some modifications. One hundred microliters of suitably diluted sample extract were added to 2900 μL of the FRAP reagent obtained by mixing acetate buffer (300 mM, pH 3.6), TPTZ (2,4,6-tripyridyl-s-triazine), 10 mM solubilized in HCl 40 mM and FeCl_3_ 20 mM in the ratio 10:1:1. The absorbance change was followed at 593 nm for 6 min. A calibration plot based on FeSO_4_·7H_2_O was used, and results were expressed as mmols of Fe^2+^ per gram of defatted and dry weight.

### 2.9. Statistical Analyses

All determinations were done in triplicate, except where differently indicated. Means and relative standard deviations were calculated. Analysis of variance (ANOVA) was performed to test the significance of the effects of the factor variables (processing steps); differences among means were separated by the least significant differences (LSD) test. Statistical analysis of data was performed using XLSTAT software version 2019.1 for Microsoft Excel (Addinsoft, New York, NY, USA). All results were considered statistically significant at *p* < 0.05.

The multivariate descriptive analysis was used to understand the presence of the main descriptors related to the BAs content of cocoa. The principal components analysis (PCA) started with the analysis of a matrix (18 × 55) that consisted of 18 samples of Criollo cocoa. The analyses were performed in triplicate. The 55 conformations of the values of the evaluated variables were gathered by the following tests: roasting temperature (raw, 120 °C for 22 min and 135 °C for 15 min), pH, content of total polyphenols (TPC), anthocyanins, antioxidant activity (FRAP and TEAC), flavonols (catechin and epicatechin), levels of the main microorganisms groups in cocoa beans and shell, ethylamine (ETH), dopamine (DOP), 2-phenylethylamine (PHE), putrescine (PUT), cadaverine (CAD), serotonin (SER), histamine (HIS), tyramine (TYR), spermidine (SPD), and spermine (SPM).

## 3. Results

### 3.1. Characterization of Fermented and Dried Cocoa Beans

Several indicators are used to measure the quality of cocoa beans. These include, in addition to microbiota, composition, colour and acidity of the beans [26].

#### 3.1.1. Microbiota

**Figure 1:**
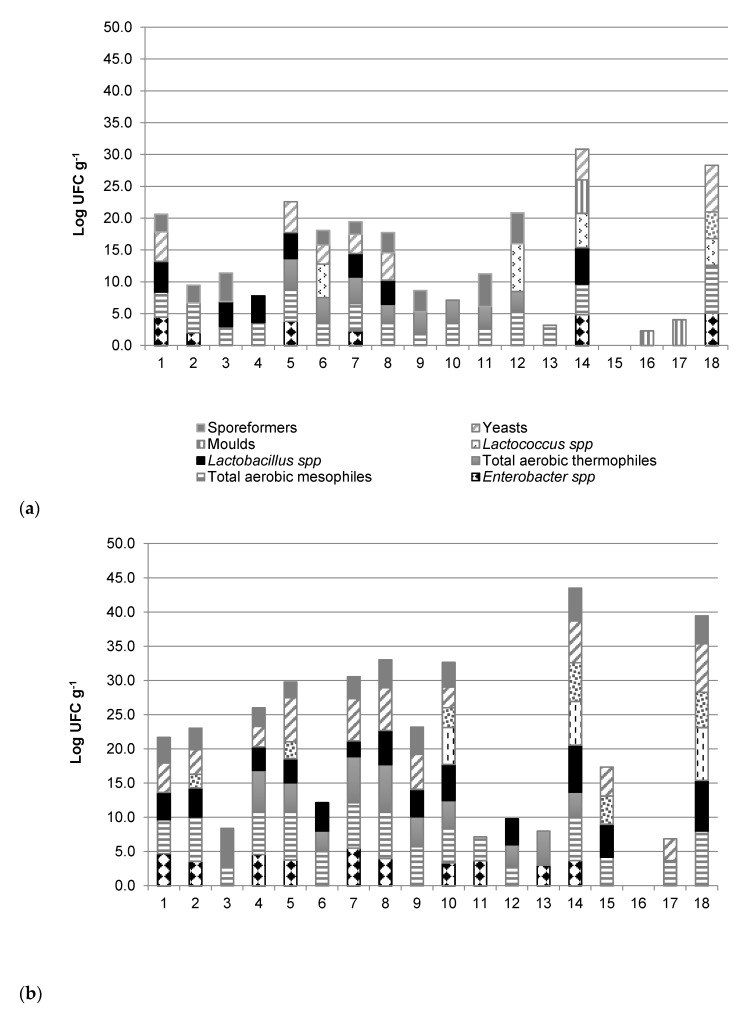
Levels of the main microorganisms groups in beans (**a**) and shells (**b**) of Colombian Criollo cocoa samples (after fermentation and drying, step T1).

Some researchers found that variations in the content of BAs in cocoa are mainly affected by fermentation, which is directly correlated to the type and quantity of microbial populations [11]. Figure 1a,b shows the distribution of microorganism groups enumerated both in cocoa beans and cocoa shells of investigated samples. A large variability was observed, confirming that postharvest practices carried out in the different Colombian farms affected microbiota, which in turn can explain the great diversity of decarboxylation products, such as BAs content at the T1 step. The microbial load of the shell was determined because many of the metabolites produced in the shell during fermentation and drying can migrate to the beans, causing a pH decrease due to the accumulation of organic acids. The microbiological analyses showed the presence of enterobacteria, total aerobic mesophiles, total aerobic thermophiles, acetic bacteria, spore-forming bacteria, lactobacilli, lactococci, fungi, and yeasts that are mainly involved in fermentation and drying. Variations in microbial counts and species were observed in the different samples, likely due to different fermentation and drying practices (pod ripeness, postharvest pod storage, variations in pulp/bean ratio, fermentation method, batch size, frequency of bean mixing or turning, and fermentation time), as well as due to some characteristics of the environment where the cultivation takes place (farm, weather conditions, pod diseases) [27,28].

A great difference was also observed between the microbial community found in the shell and inside the beans. As expected, the shell contained a greater number of microorganisms because sugars and other rapidly degradable nutrients are concentrated here, while a smaller number of microbial populations could adapt to the conditions of the beans. According to Lima et al. [29], average levels of total aerobic microorganisms and aerobic total spores are reduced in the beans, while Enterobacteriaceae and fungi were not detected.

Generally, the production of BAs is attributed to certain species of Enterobacteriaceae, mainly *Clostridium* spp., *Lactobacillus* spp., *Streptococcus* spp., *Micrococcus* spp., and *Pseudomonas* spp. [30]. Two of these groups were found in cocoa samples; Enterobacteriaceae were counted in a few samples with average values of 10^3^ CFU g^−1^, mainly in the shell and probably due to contamination during outdoor drying, while *Lactobacillus* spp. was observed in almost all the samples, with the highest count in the shell with average values of 10^4^ CFU g^−1^.

#### 3.1.2. pH, Moisture and Colour

The characteristics of Colombian Criollo cocoa samples at the end of fermentation and drying (T1) are shown in Table 2. The great variability found in the samples depends on several factors, namely fermentation and drying, as well as some intrinsic characteristics of the farming system. The organic acids produced by lactic and acetic bacteria during fermentation diffuse within the beans and cause a pH decrease; low pH values are considered an index of appropriate fermentation while pH values above 5.5 may indicate an inadequate or incomplete fermentation [31]. The pH of the samples ranged between 4.43 and 6.17 (C.V. 10%). With the exception of sample 18, it can be stated that the samples coming from Valle de Cauca were generally characterized by lower pH values compared to Cauca samples.

The moisture in bean samples ranged between 1.2% (sample 6) and 6.2% (sample 14), with differences depending on process conditions (solar or artificial dryers) and processing time. However, for all the samples, moisture content was below 12% which is considered the threshold value for optimal beans storage, corresponding to inhibition of both enzymatic reactions and fungal growth that can produce undesired metabolites during storage, such as mycotoxins.

The lightness (L*) of the 18 samples had a mean value of 40.8 (±4.03), ranging from 48.92, observed in sample 2, to 32.96 in sample 18. For redness values (a*), a mean of 7.78 (±1.94) was detected with 10.04 as the maximum value (in sample 13) and 3.64 as the minimum (in sample 2). For yellowness (b*), we observed the highest value in sample 13 (11.11 ± 2.07) and the lowest value in sample 6 (6.70 ± 0.54). Finally, for hue angle (h°), a mean value of 55.12 ± 8.26 was determined with a range from 68.77 (sample 2) to 36.49 (sample 6). Other authors reported L* values quite different from those obtained in the present study, while results for parameters of a* and b* were similar [32]. The values obtained for h° were similar to those reported by Sacchetti et al. [14].

### 3.2. Biogenic Amines Profile

The BAs profile of the Criollo cocoa beans under investigation is described in Table 3. In unroasted samples (T1), the total BAs amount was found to be 57.5 (±37.5) mg kg^−1^_DFW_, with a minimum value of 4.9 mg kg^−1^_DFW_ (in sample 16, from Nariño region) and a maximum of 127.1 mg kg^−1^_DFW_ (in sample 4, from Valle de Cauca). As far as the BAs pattern is concerned, the most represented BAs in unroasted beans (T1) were CAD, SER, HIS, SPD, and SPM (Table 3); DOP was also detected in unroasted sample 15 (from Cauca).

The Pearson correlation coefficient between total BAs content and each single BA was calculated. A strong correlation was only found with HIS (ß = 0.75); tot BAs correlated weakly with SPD (ß = 0.58), SPM (ß = 0.50), CAD (ß = 0.47), and SER (ß = 0.40), while no significant correlation was found with other amines.

To the best of our knowledge, there are no studies reporting the occurrence of CAD and HIS in raw cocoa beans, although there are few studies where BAs are identified in cocoa. Some authors [12] found tyramine, 2-phenylethylamine, tryptamine, serotonin, and dopamine in different varieties of raw cocoa beans; other authors [11] also found spermidine and spermine in Brazilian samples during fermentation.

Most of the analysed samples presented similar profiles of BAs that might be explained by the fact that they belong to the same variety. However, variations in their concentration were found and can be explained by the difference between cultivars, different growth, fermentation and drying conditions, as well as the microbiota of beans and shell (see Figure 1a,b).

Polyamines can also occur naturally due to the large proliferation of cells that occur in the early stages of growth or germination caused by physiological changes in tissues [33,34]. In fact, being that the cocoa bean is a seed and germination does not start if the optimal fermentation conditions are not present, a consequence of this could be that secondary metabolites such as the aliphatic amines (PUT, CAD, SPM and SPD) could be accumulated in cells. To our best knowledge, very few studies have been published on the relation between the physiological conditions and the BAs content in cocoa seeds, thus these aspects should be thoroughly investigated.

Although the development of microorganisms with amino acid decarboxylases activity occurs in environments with optimal pH between 4.0 and 5.5, no correlation was observed between BAs content and low pH.

No direct relationship was found between the content of polyphenols and the content of BAs in cocoa beans (see below). However, it is possible to hypothesize that the presence of metabolites as polyamines may influence antioxidant activity in cocoa samples or exhibit pro-oxidant properties [35].

### 3.3. Effect of Roasting on the BAs Content

A significant effect of both the roasting processes on total BAs content was found in all the samples; in particular, the beans treated at T2 (120 °C for 22 min) showed an increase of 60% with respect to the raw beans samples (T1), whilst the roasting process T3 (135 °C, 15 min) caused an increase of 21% compared to T1 samples.

In our experiments, we observed a large variability in the behaviour of each BA in the samples (Table 3); after the high temperature treatment, we determined the presence of TYR, 2-PHE, ETH, and PUT that were not detected in unroasted beans (T1). On the other hand, the roasting treatment increased the concentration of DOP and SPM with the increase of the temperature, while CAD and SPD levels decreased dramatically.

Several factors could affect the final accumulation of BAs. In particular, some authors have reported that Strecker degradation is responsible for the formation of BAs during the thermal decarboxylation of amino acids in the presence of α-dicarbonyl compounds formed during the Maillard reaction [12,36] or lipid peroxidation [37].

After treatment at 120 °C (T2), total BAs concentration correlated significantly with SPM (ß = 0.77), SPD (ß = 0.67) and PUT (ß = 0.60), while at 135 °C (T3) there was a strong correlation between tot BAS and SPD (ß = 0.85), HIS (ß = 0.81), and PUT, CAD and SPM (ß > 0.70).

Some authors suggested that serotonin could be formed as a result of the transformation of its precursors (tryptophan and 5-hydroxytryptophan) at very high temperatures [38]. In this study, we detected an increase in the concentration of serotonin only in three samples after T2 treatment, while this monoamine neurotransmitter in most of the samples decreased considerably after roasting at 135 °C (T3) with respect to unroasted samples (T1); a similar behaviour was observed for histamine in 50% of investigated samples. These results are in contrast with other authors who demonstrated the histamine thermostability during cooking processes [39,40].

It was also observed that the histamine level increased in foods after frying and grilling [41]. However, other authors elucidated the mechanism by which certain cooking ingredients and common organic acids destroy histamine [42], so it could be very interesting to deepen this aspect by considering the occurrence of bio-compounds that develop following the roasting process of cocoa and their possible role in the control of the BAs levels in food.

### 3.4. Anthocyanins, Total Polyphenols and Flavanols Content

The results on the content of anthocyanins, total polyphenols and flavanols of the eighteen cocoa samples at different process steps (T1, T2 and T3) are reported in Table 4.

After fermentation and drying treatment (T1), the anthocyanin concentration was between 0.17 and 3.36 mg g^−1^_DFW_, with an average value of 1.02 mg g^−1^_DFW_; these pigments disappeared during fermentation [11], reaching low values on the sixth day of fermentation, and they are a good parameter to determine the progress or status of the fermentation. The contents found are similar to other cocoa varieties from Colombia [43], but inferior to those found in other studies conducted on Ghana cocoa varieties [44]. In unroasted samples (T1), the average content of total polyphenols was 45.50 mg GAE g^−1^_DFW_, values that are similar to other cocoa varieties planted in Colombia [43,45], with the only exception being sample 4 which presented higher contents (over 80 mg GAE g^−1^_DFW_). These are more similar to the values found in other studies carried out on varieties planted in Ghana, as well as in other varieties planted in Colombia [44,46]. It is important to point out that these results may have been affected by the fact that each single phenol shows a different response to the Folin-Ciocalteau reagent [14].

According to Carrillo et al. [45], the cocoa-producing region can have a significant effect on the total polyphenol content, as a proportional relationship was found between polyphenols content and altitude of plant crops. Their results suggest that plants grown at lower altitude accumulate more polyphenols compared to plants grown at higher altitude. In the present study, the TPC determined for sample 16 (from geographical area at 30 m.a.s.l) was lower than other cocoa samples so it seems that the theory proposed by Carillo et al. is not confirmed by our data, although this aspect would be worth investigating with a large number of samples.

Roasting did not cause a statistically significant decrease in anthocyanin content in samples from all the three regions, with the following exception: a decrease of 50%–60% was observed in roasted cocoa beans in the sample of the Narino region (sample 16) due to its highest values at the end of fermentation. The decrease in anthocyanin content is in accordance with data observed by other authors [12,43] for different roasting temperatures. Regarding the TPC, a not statistically significant increase was found from 45.50 mg GAE g^−1^_DFW_ for T1 to 55.26 mg GAE g^−1^_DFW_ (+21%) for T2 and 62.01 mg GAE g^−1^_DFW_ (+14%) for T3. However, an increase in TPC values after the roasting process is consistent with the data reported by Ioannone et al. [13]; these authors suggested that an increase in TPC is dependent on temperature and exposure time, as a series of condensation and polymerization reactions occur with the formation of complex molecules such as pro-anthocyanidins from lower molecular weight compounds such as phenols and anthocyanins. Additionally, through Maillard reactions, melanoidins can be formed from reducing sugars and free amino acids; as a consequence, melanoidins can have reducing properties that affect the response to the Folin-Ciocalteu reagent, thus causing an overestimation of the TPC values [14].

The occurrence of flavanols before and after roasting was also investigated in Criollo cocoa samples and the results are reported in Table 3. Moreover, Table S1 shows the epicatechin to catechin ratio (epi/cat) for both unroasted and roasted samples. Catechin was found in all unroasted samples (ranging from n.d. to 4.43 ± 0.13) with the exception of the samples 1, 5 and 9. Epicatechin was detected in all the samples with a maximum value of 5.7 ± 0.17 mg g^−1^ (in sample 12) and a minimum of 0.45 ± 0.01 mg g^−1^ (in sample 11). Similar catechin contents were found by Loureiro et al. in dried cocoa beans from Latin America [47].

The epi/cat ratio is a widely used index as it may be associated with the degree of cocoa processing [48,49] (Table S1). Generally, with the increase of temperature the epi/cat ratio tends to decrease due to isomerization reactions and the faster degradation of epicatechin with respect to catechin [50]. The major flavanol present in unroasted samples was (−)-epicatechin. According to Hurst et al. [51], the high temperatures may induce the epimerization of this flavanol to (–)-catechin, and (+)-catechin to (+)-epicatechin. This behaviour was noticed in many samples, even though in other cases the opposite was observed. Moreover, in many cases the ratio could not be calculated since either catechin or epicatechin was not detected. Finally, it can be said that both roasting conditions had a similar effect on flavanols.

### 3.5. Trolox Equivalent Antioxidant Capacity (TEAC) and the Ferric Reducing Antioxidant Power (FRAP) Assays

Table 5 shows the effect of the different roasting treatments on the radical scavenging activity (TEAC) and the reducing activity (FRAP) assays with respect to unroasted cocoa bean samples. The Pearson correlation coefficient was calculated: a strong correlation was found between TPC content and TEAC (ß = 0.88, *p* < 0.05) and between TPC content and FRAP (ß = 0.92, *p* < 0.05).

Regarding the reducing capacity as evaluated by FRAP assay (Table 5), a mean value of 395 µmol Fe^2+^/g was determined in unroasted samples, which is in agreement with other authors [4,52].

The trend of values of FRAP was similar to those obtained in the ABTS assay. Moreover, in roasted samples results of TEAC and FRAP were comparable with values found by Ioannone et al. [13]. Generally, the antioxidant activity was higher in roasted samples compared with unroasted ones, with the exception of some cases (samples 3, 4, 7, 11, 14 and 18).

The improvement in the antioxidant and reducing properties after the roasting process may be related to the formation of reducing molecules, not quantified in the present work, as well as to the occurrence of condensation reactions among polyphenols, as evidenced by the results reported in Table 4.

### 3.6. Principal Component Analysis

**Figure 2:**
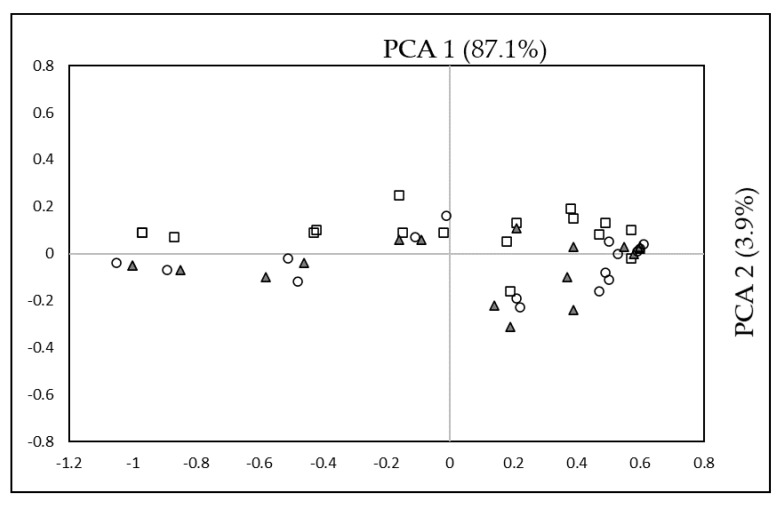
Principal component analysis related to the content of biogenic amines in Colombian criollo cocoa samples. Legend: empty square, T1 (raw cocoa beans); gray triangle, T2 (120 °C for 22 min)**;** empty circle, T3 (135 °C for 15 min).

A principal component analysis (PCA) was performed to highlight how factors or variables can influence the changes in the BA level in raw cocoa beans after roasting. Figure 2 shows the distribution of the variables analysed in the two first principal components which represent 90.1% of data variance. Usually, the two first principal components are sufficient to explain the maximum variation in all data [31]. PC1 and PC2 explained 84.9% and 5.2% of date variance related to the BA content of cacao. In order to better describe the data set, the following results and information were included: microbial counts, polyphenols (TPC), anthocyanins, antioxidant activity (FRAP and TEAC), flavonols (catechin and epicatechin), BA content at different processing conditions (T1, T2, and T3) and origin of the samples.

Concerning PC1, antioxidant activity (FRAP and TEAC) (−1.23 to −1.01), polyphenol content (TPC), and BA (−0.72 to −0.56) showed a negative influence on this component, while FRAP, TEAC, and TPC showed a significant increase in concentration in the same way as BA during roasting conditions. On the other side, anthocyanins, catechin, and epicatechin (0.43 to 0.55) showed a positive influence on this component. The anthocyanin content is a good parameter to determine if fermentation is carried out properly since they decrease as the fermentation progresses; therefore, the correlation found between a high anthocyanin content in raw cocoa (T1) and a high BA content may be related to a non-ideal fermentation process in which, for different reasons, the enzymatic activity of the grains remained active, generating metabolic intermediates such as BAs.

As for the individual BAs, a positive influence was found for DOP (0.51) under initial conditions (T1) after treatment at 120 °C (T2) for PUT, CAD, and SPD (0.49 to 0.54), and at 135 °C (T3) for CAD, SPD, SER, HIS, and SPM (0.44 to 0.56), while the other BAs showed no influence on this component.

The variables pH (0.1), region (0.41), shell microbiota (−0.26), and bean microbiota (−0.12) showed a weak correlation with each other. The pH is important to select the type of microorganisms that can grow and therefore quantity, and on the type of BAs they can generate [53]; in the present study, the values found for pH were not low enough to inhibit Enterobacteriaceae, which is one of the main groups that can produce BAs [31]. Moreover, pH values were in the optimal range that can favour BAs accumulation. The synthesis of polyamines, such as spermine and spermidine, occurs in response to high pH environments; these BAs act as inhibitors of carbonic anhydrase enzymes that catalyse the interconversion of carbon dioxide and water into bicarbonate and protons and vice versa [54].

According to Lima et al. [29], average levels of microorganisms are lower in the beans compared to those found in the shell due to the lower availability of nutrients, which can cause the activation of metabolic pathways in some groups of microorganisms that can lead to the accumulation of decarboxylation products such as BAs; however, no influence was observed in this component. Regarding the origin, the difference between cultivars, different growth, and postharvest conditions may be related to the presence of these BAs, but no influence in this component was established between the different sites where the samples were taken.

Concerning PC2, this component was mainly influenced by roasting. On the positive axis, the characteristics of the beans without heat treatment (T1) were located predominantly, differing from the samples T2 and T3 that were located on the negative axis of the component. Although a statistically significant difference was found in the content of BAs at T1, T2 and T3 in most of the samples, in the PC2 component only a small correlation was evident among them.

## 4. Conclusions

The present study aimed to evaluate the accumulation of bioactive compounds in eighteen Criollo cocoa beans samples from Colombia, with a special focus on biogenic amines and polyphenols, after fermentation and drying and after two different roasting processes commonly used in cocoa factories.

The samples showed a similar BAs profile, with a variability in their concentration as a consequence of both cocoa beans and shell microbiota, as well as differences among cultivars, growth conditions and fermentation and drying treatments. High temperature seems to correlate with the occurrence of TYR, PHE, ETH and PUT; moreover, the roasting process significantly increased the concentration of DOP and SPM, whilst CAD and SPD levels generally decreased. The total phenolic content was positively affected by the roasting processes; even without a statistically significant difference a remarkable improvement in the antioxidant and reducing properties were observed, showing an enhancement of their functionality.

No direct relationship was found between the content of polyphenols and the content of BAs in cocoa beans, even if it can be speculated that polyamines could have a role by influencing the antioxidant activity or exhibiting pro-oxidant properties in cocoa beans. Therefore, the correlation found between a high anthocyanin content and a high BAs content in unroasted cocoa samples (T1) could be attributable to a non-ideal fermentation process. One important result that it is worth pointing out is that the quantities of BAs found in the unroasted cocoa beans were not alarming, especially with regard to HIS and TYR, the amines of toxicological interest.

Moreover, low BAs amounts were also found in roasted samples, which is of crucial importance considering that such values that were calculated for defatted samples will be further processed and used as ingredients in complex formulations.

## Figures and Tables

**Table 1:** Geographical characteristics of the area of origin and fermentation and drying condition of the cocoa samples.

	Farm Location	Altitude (masl *)	Fermentation				Drying			
			T_day_ (°C)	T_night_ (°C)	Time (d)	Box	T_day_ (°C)	T_night_ (°C)	Time (d)	Drying Surface
1	Valle del Cauca	1000	27–31	18–20	4	plastic	28–30	17–18	4	wooden trays
2	Valle del Cauca	1000	27–31	18–20	3	plastic	28–30	17–18	5	wooden trays
3	Valle del Cauca	1000	25–27	17–18	6	wooden	25–27	18–19	3	wooden trays
4	Valle del Cauca	1000	27–31	18–20	6	plastic	28–30	17–18	3	wooden trays
5	Cauca	990	29–30	19–20	4	wooden	29–30	18–19	5	wooden trays
6	Valle del Cauca	1000	27–31	18–20	6	wooden	28–30	17–18	6	floors
7	Valle del Cauca	1000	25–27	17–18	6	plastic	25–27	18–19	4	wooden trays
8	Valle del Cauca	1000	25–27	17–18	6	plastic	25–27	18–19	4	metal trays
9	Cauca	990	29–30	19–20	4	wooden	29–30	18–19	6	floors
10	Cauca	990	28–33	19–20	4	wooden	28–33	17–20	7	floors
11	Valle del Cauca	1000	27–31	18–20	6	wooden	28–30	17–18	4	floors
12	Valle del Cauca	1000	27–31	18–20	6	plastic	28–30	17–18	3	floors
13	Valle del Cauca	1000	25–27	17–18	6	plastic	25–27	18–19	3	wooden trays
14	Cauca	990	29–30	19–20	4	wooden	29–30	18–19	5	wooden trays
15	Valle del Cauca	1000	27–31	18–20	6	plastic	28–30	17–18	5	wooden trays
16	Nariño	30	21–25	12–22	4	wooden	20–25	11–15	4	wooden trays
17	Valle del Cauca	1000	27–31	18–20	6	plastic	28–30	17–18	4	floors
18	Valle del Cauca	1000	25–27	17–18	6	plastic	25–27	18–19	3	wooden trays

* meters above sea level.

**Table 2:** Chemico-physical and colour parameters (L*, a*, b* and h°) for fermented and dried samples (T1). The data are expressed as mean ± standard deviation.

Samples	Origin	pH	Moisture (%)	Colour			
				L*	a*	b*	h°
1	Valle de Cauca	5.01 ± 0.02	3.4 ± 0.3	40.83 ± 0.80	7.06 ± 0.08	11.17 ± 0.35	57.67± 0.01
2	Valle de Cauca	4.79 ± 0.11	3.9 ± 0.2	48.92 ± 0.27	3.64 ± 0.08	9.38 ± 0.10	68.77 ± 0.02
3	Valle de Cauca	4.43 ± 0.08	4.2 ± 0.4	41.78 ± 0.43	9.87 ± 0.12	12.35 ± 0.22	51.35 ± 0.06
4	Valle de Cauca	4.49 ± 0.03	3.8 ± 0.3	38.40 ± 0.16	9.49 ± 0.12	9.57 ± 0.13	45.24 ± 0.02
5	Cauca	5.85 ± 0.06	5.0 ± 0.2	41.42 ± 0.30	9.21 ± 0.05	10.25 ± 0.16	48.07 ± 0.01
6	Valle de Cauca	4.54 ± 0.10	1.2 ± 0.1	38.76 ± 0.67	9.05 ± 0.32	6.70 ± 0.54	36.49 ± 0.02
7	Valle de Cauca	4.99 ± 0.07	3.5 ± 0.2	41.71 ± 0.65	8.29 ± 0.15	10.85 ± 0.16	52.62 ± 0.01
8	Valle de Cauca	5.05 ± 0.05	4.8 ± 0.4	43.26 ± 0.37	8.25 ± 0.27	13.43 ± 0.20	58.45 ± 0.01
9	Cauca	5.11 ± 0.18	2.5 ± 0.1	36.84 ± 0.30	7.30 ± 0.16	11.79 ± 0.41	58.22 ± 0.01
10	Cauca	5.52 ± 0.15	1.7 ± 0.1	38.89 ± 0.68	4.37 ± 0.02	8.30 ± 0.09	62.22 ± 0.01
11	Valle de Cauca	4.41 ± 0.08	2.5 ± 0.2	39.85 ± 0.34	9.60 ± 0.24	10.27 ± 0.38	46.90 ± 0.01
12	Valle de Cauca	4.62 ± 0.03	2.2 ± 0.0	44.50 ± 0.38	7.31 ± 0.16	11.34 ± 0.18	57.19 ± 0.00
13	Valle de Cauca	4.67 ± 0.33	4.9 ± 0.4	37.54 ± 0.18	10.04 ± 0.05	15.77 ± 0.03	57.53 ± 0.00
14	Cauca	5.45 ± 0.09	6.2 ± 0.3	38.44 ± 1.02	8.18 ± 0.09	12.07 ± 0.26	55.87 ± 0.01
15	Valle de Cauca	5.07 ± 0.12	2.5 ± 0.2	38.23 ± 0.91	8.33 ± 0.14	9.30 ± 0.08	48.15 ± 0.00
16	Nariño	4.68 ± 0.13	3.9 ± 0.1	43.78 ± 0.07	5.34 ± 0.09	12.25 ± 0.25	66.43 ± 0.01
17	Valle de Cauca	4.44 ± 0.10	2.8 ± 0.2	48.89 ± 0.73	5.34 ± 0.09	12.25 ± 0.25	66.43 ± 0.01
18	Valle de Cauca	6.17 ± 0.27	4.7 ± 0.7	32.96 ± 0.89	9.39 ± 0.32	12.93 ± 0.46	54.00 ± 0.00

**Table 3:** Biogenic amines (mg kg^−1^_DFW_) in Criollo cocoa bean samples after fermentation and drying (T1) and after roasting (T2, 120 °C for 22 min; T3, 135 °C for 15 min).

Sample		Biogenic Amines Content (mg kg^−1^_DFW_)									
		ETH *	DOP	PHE	PUT	CAD	SER	HIS	TYR	SPD	SPM
1	T1	nd	nd	nd	nd	49.75 ± 1.4	3.06 ± 0.0	5.96 ± 1.2	nd	30.85 ± 3.6	nd
	T2	14.72 ± 2.3	nd	12.2 ± 0.1	1.32 ± 0.2	nd	17.18 ± 4.2	0.21 ± 3.1	17.02 ± 0.2	nd	14.30 ± 2.2
	T3	15.56 ± 5.2	nd	26.23 ± 0.3	59.02 ± 3.5	5.38 ± 0.6	nd	13.25 ± 1.1	11.11 ± 0.0	nd	36.84 ± 5.2
2	T1	nd	nd	nd	nd	48.62 ± 0.2	0.60 ± 0.0	nd	nd	4.67 ± 0.3	nd
	T2	24.77 ± 0.7	33.27 ± 0.2	11.18 ± 0.1	nd	nd	17.70 ± 0.0	nd	26.51 ± 4.2	nd	46.42 ± 8.4
	T3	19.22 ± 0.4	1.99 ± 0.0	25.98 ± 1.2	62.58 ± 6.2	nd	2.95 ± 0.2	17.13 ± 0.9	11.27 ± 0.8	2.50 ± 0.0	52.82 ± 3.9
3	T1	nd	nd	nd	nd	nd	nd	3.56 ± 1.1	nd	13.91 ± 0.2	0.37 ± 0.0
	T2	0.89 ± 0.3	nd	12.55 ± 0.5	nd	nd	9.11 ± 0.6	0.44 ± 0.5	25.22 ± 1.7	nd	nd
	T3	1.08 ± 0.4	nd	9.69 ± 136	nd	nd	nd	0.80 ± 1.2	8.29 ± 0.3	nd	nd
4	T1	nd	nd	nd	nd	22.60 ± 0.6	11.65 ± 1.1	41.9 ± 2.2	nd	42.06 ± 2.8	8.86 ± 3.2
	T2	2.74 ± 0.1	nd	11.77 ± 0.1	nd	2.22 ± 0.1	3.07 ± 0.0	0.82 ± 0.5	14.96 ± 1.1	nd	2.03 ± 1.8
	T3	1.68 ± 0.2	nd	17.22 ± 0.0	nd	nd	1.01 ± 0.0	1.01 ± 1.2	15.98 ± 0.4	nd	nd
5	T1	nd	nd	nd	nd	66.57 ± 0.7	0.36 ± 0.0	39.8 ± 7.1	nd	5.99 ± 0.0	nd
	T2	29.17 ± 0.3	65.12 ± 0.1	10.36 ± 0.5	6.46 ± 0.3	3.28 ± 0.0	25.63 ± 2.3	0.46 ± 0.7	20.80 ± 0.5	nd	60.04 ± 8.4
	T3	17.06 ± 1.2	95.03 ± 0.1	10.67 ± 0.1	2.51 ± 0.1	3.87 ± 0.0	nd	0.38 ± 0.2	9.87 ± 0.0	1.15 ± 0.0	nd
6	T1	nd	nd	nd	nd	1.50 ± 0.0	6.98 ± 0.1	38.90 ± 5.3	nd	48.66 ± 2.1	13.61 ± 2.2
	T2	2.95 ± 1.2	nd	11.36 ± 0.1	nd	3.14 ± 0.0	nd	17.13 ± 2.2	17.03 ± 0.7	nd	5.38 ± 0.4
	T3	2.17 ± 0.7	nd	14.75 ± 0.1	nd	nd	nd	nd	13.50 ± 0.4	nd	nd
7	T1	nd	nd	nd	nd	1.80 ± 0.0	0.11 ± 0.0	5.96 ± 1.2	nd	18.53 ± 6.2	1.12 ± 0.0
	T2	0.98 ± 0.1	nd	13.85 ± 0.0	nd	nd	nd	13.55 ± 3.0	20.51 ± 0.2	nd	nd
	T3	1.24 ± 0.1	nd	15.72 ± 0.2	nd	nd	nd	nd	13.12 ± 0.8	nd	nd
8	T1	nd	nd	nd	nd	15.86 ± 0.1	nd	nd	nd	12.81 ± 2.5	0.12 ± 0.1
	T2	3.61 ± 0.4	nd	12.70 ± 0.1	nd	nd	nd	48.18 ± 1.9	14.88 ± 0.1	nd	1.05 ±
	T3	3.36 ± 0.3	nd	12.81 ± 0.1	nd	nd	nd	nd	10.82 ± 0.0	nd	nd
9	T1	nd	nd	nd	nd	51.77 ± 0.5	0.85 ± 0.1	nd	nd	11.93 ± 1.1	0.37 ± 0.3
	T2	13.67 ± 0.5	nd	14.14 ± 0.1	nd	nd	nd	37.73 ± 2.8	18.16 ± 7.7	0.51 ± 0.0	nd
	T3	11.3 ± 0.1	nd	14.98 ± 0.1	1.50 ±	2.31 ± 0.0	nd	nd	15.85 ± 1.6	0.03 ± 0.0	nd
10	T1	nd	nd	nd	nd	nd	nd	7.76 ± 4.1	nd	12.37 ± 0.0	2.37 ± 0.2
	T2	21.65 ± 0.1	76.92 ± 0.1	12.04 ± 0.1	3.99 ± 0.4	2.22 ± 0.0	3.71 ± 0.1	0.27 ± 0.7	14.97 ± 1.1	1.87 ± 0.0	nd
	T3	12.55 ± 0.2	56.68 ± 0.1	8.42 ± 0.5	nd	nd	nd	1.25 ± 0.2	11.5 ± 2.7	nd	nd
11	T1	nd	nd	nd	nd	5.54 ± 0.1	0.24 ± 0.0	5.04 ± 0.1	nd	23.15 ± 1.2	4.37 ± 0.6
	T2	2.63 ± 0.1	nd	13.35 ± 0.0	nd	nd	nd	21.53 ± 3.1	14.20 ± 0.7	nd	nd
	T3	2.89 ± 0.1	nd	14.83 ± 0.6	nd	nd	nd	0.27 ± 0.5	14.49 ± 5.7	nd	0.37 ± 0.2
12	T1	nd	nd	nd	nd	7.56 ± 0.1	4.28 ± 0.1	16.74 ± 3.1	nd	44.48 ± 4.2	9.61 ± 1.1
	T2	4.55 ± 0.4	nd	13.94 ± 3.2	nd	nd	nd	59.78 ± 6.4	19.68 ± 0.3	nd	nd
	T3	3.19 ± 0.2	nd	16.16 ± 4.1	nd	nd	nd	1.24 ± 0.2	8.54 ± 0.6	nd	nd
13	T1	nd	nd	nd	nd	0.61 ± 0.0	4.41 ± 1.1	4.76 ± 2.6	nd	40.75 ± 2.7	4.86 ± 0.2
	T2	8.38 ± 0.1	nd	11.81 ± 2.9	nd	nd	nd	0.60 ± 0.3	16.24 ± 0.5	nd	nd
	T3	7.46 ± 0.1	nd	13.43 ± 1.6	nd	nd	nd	0.17 ± 0.4	14.42 ± 0.8	nd	nd
14	T1	nd	nd	nd	nd	nd	15.33 ± 1.2	2.96 ± 0.3	nd	18.31 ± 3.1	0.62 ± 0.1
	T2	6.47 ± 0.2	nd	14.65 ± 2.8	nd	nd	5.62 ± 0.1	0.79 ± 0.7	18.51 ± 1.0	nd	nd
	T3	6.33 ± 0.3	nd	12.50 ± 3.1	nd	2.87 ± 0.0	nd	2.70 ± 0.5	12.41 ± 3.4	nd	nd
15	T1	nd	57.35 ± 01	nd	nd	0.83 ± 0.0	3.55 ± 0.0	nd	0.19 ± 0.0	24.47 ± 2.2	8.86 ± 0.8
	T2	11.92 ± 0.0	nd	16.27 ± 2.1	nd	nd	4.90 ± 0.0	nd	13.65 ± 0.3	nd	nd
	T3	6.102 ± 0.2	nd	10.81 ± 1.0	nd	nd	nd	nd	12.14 ± 0.6	nd	nd
16	T1	nd	nd	nd	nd	2.85 ± 0.0	1.34 ± 0.0	nd	nd	0.28 ± 0.0	0.42 ± 0.0
	T2	27.00 ± 0.2	92.17 ± 0.1	18.78 ± 1.2	nd	nd	nd	29.87 ± 5.6	9.64 ± 0.0	2.62 ± 0.2	3.35 ± 0.7
	T3	9.41 ± 0.8	55.76 ± 0.1	6.02 ± 0.0	nd	nd	nd	1.18 ± 9.4	0.79 ± 0.0	nd	nd
17	T1	nd	nd	nd	nd	5.77 ± 0.3	9.68 ± 0.4	5.36 ± 0.7	nd	14.79 ± 2.1	1.19 ± 0.5
	T2	23.52 ± 0.4	77.52 ± 0.1	11.81 ± 0.8	nd	5.45 ± 0.2	nd	27.27 ± 4.9	11.37 ± 0.4	2.30 ± 0.0	nd
	T3	22.27 ± 0.0	16.27 ± 0.1	11.73 ± 0.3	4.44 ± 1.1	5.45 ± 0.7	nd	8.12 ± 0.9	12.51 ± 0.0	2.60 ± 0.0	2.85 ± 0.0
18	T1	nd	nd	nd	nd	nd	nd	nd	nd	15.45 ± 0.0	0.87 ± 0.0
	T2	25.66 ± 0.1	nd	17.24 ± 2.1	nd	nd	nd	nd	15.14 ± 3.2	nd	nd
	T3	11.77 ± 0.1	nd	20.60 ± 1.6	nd	nd	nd	nd	16.94 ± 1.9	nd	nd

* Legend: ethylamine (ETH), dopamine (DOP), 2-phenylethylamine (PHE), putrescine (PUT), cadaverine (CAD), histamine (HIS), serotonin (SER), tyramine (TYR), spermidine (SPD), and spermine (SPM); nd, not detectable.

**Table 4:** Anthocyanins, total polyphenols (TPC) and flavanols content of the Criollo cocoa samples after fermentation and drying (T1) and after roasting (T2, 120 °C for 22 min; T3, 135 °C for 15 min). The data are expressed as mean of triplicate analysis.

Sample	Anthocyanins (mg g^−1^_DFW_)			Sign.	TPC (mg GAE g^−1^_DFW_)			Sign.	Catechin (mg g^−1^_DFW_)			Sign.	Epicatechin (mg g^−1^_DFW_)			Sign.
	T1	T2	T3		T1	T2	T3		T1	T2	T3		T1	T2	T3	
1	2.28^a^	1.36^c^	2.08^b^	*	43.00^c^	68.79^b^	83.85^a^	*	nd	8.41^b^	11.49^a^	**	0.5^b^	0.87^a^	0.51^b^	*
2	3.36^a^	1.77^b^	2.00^b^	*	22.97^b^	100.73^a^	110.17^a^	***	0.18^b^	4.36^a^	nd	***	0.76	0.9	0.54	**
3	0.24^c^	0.31^b^	0.37^a^	*	47.38^b^	33.54^a^	45.56^b^	*	1.47^b^	1.64^b^	2.17^a^	*	1.72^a^	nd	0.7^b^	**
4	0.76^a^	0.59^b^	0.51^b^	*	85.75^a^	48.56^c^	56.42^b^	*	1.82^b^	2.23^b^	3.72^a^	*	1.41	nd	nd	*
5	2.33^a^	1.79^b^	1.80^b^	*	40.66^b^	79.91^a^	83.76^a^	*	n.d	3.36^b^	4.66^a^	*	1.16^a^	nd	1.1^a^	*
6	0.36^b^	0.50^a^	0.56^c^	*	65.12^a^	68.36^a^	60.39^b^	*	2.16^b^	5.93^a^	6.12^a^	**	1.07^a^	0.47^b^	nd	*
7	0.35^b^	0.33^b^	0.45^a^	*	70.61^a^	33.29^b^	44.41^b^	*	0.16^b^	1.69^a^	0.16^b^	**	1.29	nd	nd	*
8	0.29^c^	0.33^b^	0.45^a^	*	34.04^b^	29.33^b^	43.24^a^	*	0.49^a^	0.17^b^	0.18^b^	*	1.22	nd	nd	*
9	1.42^a^	0.97^c^	0.86^b^	*	47.34^c^	83.17^a^	57.77^b^	*	nd	0.65	nd	*	1.26	nd	nd	*
10	1.94^c^	1.86^b^	2.29^a^	*	54.73^c^	75.15^b^	89.98^a^	*	0.03^b^	13.02^a^	0.19^b^	***	0.45^b^	nd	0.83^a^	*
11	0.57^a^	0.49^b^	0.52^ab^	*	53.02^a^	40.38^c^	46.23^b^	*	4.35^a^	3.06^b^	0.13^c^	*	5.7^a^	nd	3.58^b^	**
12	0.46	0.48	0.44	n.s.	49.59^b^	55.46^a^	58.36^a^	**	3.42^b^	4.39^a^	0.42^c^	*	1.61^b^	nd	5.26^a^	***
13	0.17^c^	0.23^b^	0.31^a^	*	22.62^b^	18.99^b^	38.49^a^	*	4.43^a^	0.74^b^	0.05^c^	*	0.59^b^	nd	2.07^a^	**
14	0.92^b^	0.48^c^	1.15^a^	*	42.02^b^	28.78^c^	48.02^a^	*	0.66	0.13	0.33	n.s.	0.79^b^	nd	2.62^a^	**
15	0.52^b^	0.64^a^	0.66^a^	*	32.90^c^	46.56^b^	57.72^a^	**	4.43^b^	6.61^a^	nd	**	2.34^a^	1.62^b^	nd	**
16	1.39^a^	0.63^c^	0.84^b^	*	23.89^b^	60.64^a^	62.91^a^	**	4.00^b^	0.50^b^	nd	**	0.97^b^	5.87^a^	0.78^b^	***
17	0.72^a^	0.71^a^	0.35^b^	*	33.83^b^	87.43^a^	88.57^a^	***	0.65^b^	0.27^b^	nd	*	0.7^c^	2.27^a^	1.14^b^	*
18	0.35^b^	0.52^a^	0.60^a^	*	49.53^a^	35.56^c^	40.32^b^	*	0.09^b^	0.11^b^	1.65^a^	*	1.58^a^	0.95^b^	0.53^c^	*

Legend: nd, not detectable; data followed by different superscript letters, in the same line, are significantly different (LSD test, *p* < 0.05); asterisks indicate significance at * *p* < 0.05; ** *p* < 0.01; *** *p* < 0.001; n.s. not significant.

**Table 5:** Results of Trolox Equivalent Antioxidant Capacity (TEAC) and the Ferric Reducing Antioxidant Power (FRAP) assays on the Criollo cocoa samples after fermentation and drying (T1) and after roasting (T2, 120 °C for 22 min; T3, 135 °C for 15 min). The data are expressed as mean of triplicate analysis.

Sample	TEAC (µmol TE g^−1^)			Sign.	FRAP (µmol Fe^2+^ g^−1^)			Sign.
	T1	T2	T3		T1	T2	T3	
1	293.6^b^	270.6^c^	374.0^a^	*	374.9^c^	594.7^a^	439.4^b^	*
2	125.0^c^	529.8^b^	578.6^a^	***	144.6^c^	714.8^b^	790.6^a^	**
3	380.1^a^	100.9^c^	193.5^b^	*	382.4^a^	217.5^c^	261.9^b^	*
4	304.7^a^	181.7^b^	158.2^c^	*	486.6^a^	301.5^c^	405.6^b^	*
5	200.4^c^	313.5^b^	384.6^a^	*	249.2^b^	521.5^a^	488.0^a^	*
6	268.4^a^	220.7^b^	260.9^a^	*	510.1	481.2	488.3	n.s.
7	411.0^a^	94.5^c^	170.6^b^	***	562.3^a^	242.3^b^	243.0^b^	*
8	101.5^b^	91.4^b^	187.9^a^	*	259.2^a^	187.0^b^	269.9^a^	*
9	161.4^c^	396.9^a^	188.9^b^	*	282.6^c^	321.0^b^	431.2^a^	*
10	217.1^c^	389.0^b^	459.3^a^	*	432.2^c^	562.7^b^	703.5^a^	**
11	238.4^a^	135.2^c^	168.3^b^	*	401.9^a^	282.0^b^	279.5^b^	*
12	246.3^b^	284.8^a^	279.2^a^	*	313.8^c^	352.0^b^	440.1^a^	*
13	98.2^b^	69.6^c^	141.3^a^	**	174.5^b^	135.7^c^	222.1^a^	***
14	195.5^a^	136.1^c^	161.1^b^	*	264.3^b^	252.8^b^	334.3^a^	*
15	161.3^b^	202.5^a^	111.3^c^	*	205.6^c^	349.9^b^	466.3^a^	**
16	184.1^c^	224.8^b^	351.0^a^	*	151.1^c^	388.4^b^	498.8^a^	***
17	191.0^c^	391.9^b^	441.5^a^	***	214.6^c^	636.9^b^	789.1^a^	***
18	231.0^a^	167.2^b^	122.3^c^	*	275.3	229.1	273.8	n.s.

Legend: data followed by different superscript letters, in the same line, are significantly different (LSD test, *p* < 0.05); asterisks indicate significance at * *p* < 0.05; ** *p* < 0.01; *** *p* < 0.001; n.s. not significant.

## Supplementary Materials

The following are available online at https://www.mdpi.com/2304-8158/9/4/520/s1, Table S1: The ratio of epicatechin to catechin (epi/cat) in cocoa bean samples, fermentation and drying (T1) and after roasting (T2 and T3).
