# Supplementary material for: Effect of Fermentation, Drying and Roasting on Biogenic Amines and Other Biocompounds in Colombian Criollo Cocoa Beans and Shells
Source: Foods. 2020 Apr 21;9(4):520. doi: 10.3390/foods9040520 (PMC7231058; doi:10.3390/foods9040520)
Supplement: Supplementary file 1 [file foods-09-00520-s001.pdf]

Table S1. The ratio of epicatechin to catechin (epi/cat) in cocoa bean samples, post-harvest (T1) and after roasting treatment (T2 and T3).

samples	T1	T2	T3
1	-	0.10	0.04
2	4.22	0.20	-
3	1.17	-	0.32
4	0.77	-	-
5	-	-	0.23
6	0.49	0.08	-
7	8.06	-	-
8	2.48	-	-
9	-	-	-
10	15	-	4.36
11	1.31	-	27.53
12	0.47	-	12.52
13	0.13	-	41.4
14	1.19	-	7.93
15	0.52	0.24	-
16	0.24	11.74	-
17	1.07	8.4	-
18	17.55	8.36	0.32
